# What does it mean for consciousness to be multidimensional? A narrative review

**DOI:** 10.3389/fpsyg.2024.1430262

**Published:** 2024-06-20

**Authors:** Julie Páleník

**Affiliations:** First Department of Neurology, St. Anne’s University Hospital and Medical Faculty of Masaryk University, Brno, Czechia

**Keywords:** consciousness, global state, dimensionality, dimensions of consciousness, multidimensional properties, multidimensional phenomenon, complexity measures, structure of consciousness

## Abstract

A recent development in the psychological and neuroscientific study of consciousness has been the tendency to conceptualize consciousness as a multidimensional phenomenon. This narrative review elucidates the notion of dimensionality of consciousness and outlines the key concepts and disagreements on this topic through the viewpoints of several theoretical proposals. The reviewed literature is critically evaluated, and the main issues to be resolved by future theoretical and empirical work are identified: the problems of dimension selection and dimension aggregation, as well as some ethical considerations. This narrative review is seemingly the first to comprehensively overview this specific aspect of consciousness science.

## 1 Introduction

Consciousness is a multidimensional phenomenon. Or, at least, this is the claim of a number of recent papers in the field of consciousness research (Fazekas and Overgaard, 2016; Jonkisz et al., 2017; Bayne and Carter, 2018; Walter, 2021; Huang et al., 2023). This interest in the dimensionality of consciousness follows after the seminal work of Bayne et al. (2016a). However, to the author’s knowledge, the different viewpoints on the dimensionality of consciousness have not yet been reviewed and critically evaluated in a single text. The presented narrative review is therefore seemingly the first of its kind to provide an overview of this specific aspect of the science of consciousness, which is nonetheless of importance to all theories of consciousness.

The scope of this review is limited to the dimensionality of human consciousness, excluding consciousness of artificial systems and animals other than humans. Only models that clearly endorse a dimensional description of consciousness or some of its basic aspects are considered here. This leaves out models that may only *imply* a dimension or dimensions to consciousness, which is fairly common in theoretical texts on consciousness, as has been noted by others (Jonkisz et al., 2017; Schwitzgebel, 2023). The review is also restricted to models that draw on cognitive science and philosophy. Models rooted in religion or spirituality, as well as quantum theories of consciousness, have not been included.

The main body of the review is divided into three sections. The first one presents some general issues of multidimensional properties and their specifics in the case of the dimensionality of consciousness. The second section is divided into subsections reviewing different theoretical perspectives on the dimensionality of consciousness, grouping texts roughly according to which authors tend to publish together. Due attention is given to authors responding to others’ proposals and relating their own work to theirs. Finally, the third section briefly focuses on empirical research that has been associated with the topic of dimensions of consciousness.

The body of work reviewed here differentiates itself from consciousness research at large by presupposing that consciousness is the kind of property that can be meaningfully placed on some dimension or dimensions. This entails accepting some non-trivial premises, as well as committing to the use of dimensional language with potentially problematic semantics, as will be discussed in the next section. Therefore, if the science of consciousness is to embrace or reject the notion of a dimensional structure or dimensional description of consciousness, it cannot do so out of hand but only after a critical examination of the associated issues, which will hopefully be made easier by the review presented here.

## 2 Multidimensional properties

The noun *dimension*, in the sense relevant to our discussion, signifies an extent, magnitude, or degree of something (Oxford English Dictionary, n.d.). The derived terms *dimensional* and *multidimensional* are therefore taken to straightforwardly mean “having (multiple) dimensions.” To understand the possible issues associated with a property being (multi)dimensional, we may turn to the fields of linguistics and philosophy^1^.

Dimensional adjectives depend on the value of some scalar variable, such as *tall* depending on the height of a person (Sassoon, 2013). While their comparative forms (“is taller than”), given the correct context, are relatively unproblematic, the positive form (“is tall”) often introduces vagueness, resulting in statements with unclear truth values (Kennedy, 2007). The positive form must be true or false relative to a certain criterion; for example, it might be enough that the object has a non-zero value on the relevant dimensions for the positive form to be true. Lee (2023) endorses this so-called “minimal threshold” option for consciousness. If consciousness is gradable, having even a small amount of consciousness means being conscious.

Multidimensional adjectives, then, are dependent on multiple scalar variables. A typical example could be the adjective *healthy* in relation to values of cholesterol or blood pressure, among others (Sassoon, 2013). This raises a new problem with the comparative form: two objects could be positioned oppositely on some of the constituent dimensions, i.e., one person having a higher cholesterol but lower blood pressure than another. D’Ambrosio and Hedden (2023) propose a semantic framework for multidimensional adjectives that admits multiple valid *aggregation functions* which describe the way dimensions are combined to decide the truth value of a comparison. Vagueness arises from the fact that most multidimensional adjectives have more than one admissible aggregation function.

Lee (2023) brings up further issues when treating consciousness as a dimensional or multidimensional property. A property being multidimensional should be distinguished from a multidimensional object (e.g., a three-dimensional shape) having a property that is actually unidimensional (volume) or even non-gradable (having four sides). This problem is illustrated by a disagreement between Bayne et al. (2016b) and Fazekas and Overgaard (2016) in section 3.4 below, which concerns whether the conscious awareness of perceptual content comes in degrees.

It is also possible that consciousness varies along a very large number of dimensions (*massive multidimensionality*; Lee, 2023). In this case, even if there were only a few valid aggregation functions or if it was required for consciousnesses to differ in the same direction on all dimensions, almost no two instances of consciousness could be compared, making consciousness itself for all intents and purposes unorderable (but not its dimensions).

Another source of semantic vagueness in dimensional or multidimensional adjectives comes in the form of *borderline cases* (Égré and Zehr, 2018) when neither the positive form nor its negation are considered true (“they are neither healthy nor unhealthy”), or alternatively, when both the positive form and its negation are (“they are both healthy and unhealthy”). This specific kind of vagueness has usually been denied to the property of consciousness on philosophical grounds (Antony, 2008; Simon, 2017). However, recently, Schwitzgebel (2023) has argued in favor of the existence of borderline cases of consciousness.

To conclude, there are certain theoretical preconditions to be accepted before proceeding to claim that consciousness is (multi)dimensional. It is taken for granted that our conscious experience varies in time. The first assumption, then, is that at least some of this variation in experience is equivalent to variation in consciousness itself. The second necessary assumption is that variation in consciousness can be meaningfully organized into a continuous, graded dimension.

Rejecting the first assumption, there is only one consciousness, and all of the variation in experience translates into different mental phenomena, such as perception, attention, thought, or memory. Subjects just are conscious *simpliciter*, or they are not. Accepting the first assumption but rejecting the second one, there are different kinds of consciousness (e.g., an awake and a dreaming one), but they are not orderable on any continuous scale.

In addition, on the mainstream philosophical opinion, if consciousness is unidimensional, it cannot be vague. One would have to accept the possibility of states that are neither conscious nor unconscious (Schwitzgebel, 2023). On the other hand, if consciousness is multidimensional, there is additional room for vagueness in the global ordering of different consciousnesses. For example, it may be impossible to order some pairs of species based on which is more conscious than the other.

Here, I would also like to note that a difference could be made between what might be called dimensional *description* and the dimensional *structure* of consciousness. In the case of dimensional description, dimensions serve only as useful abstractions, making no commitments about the actual structure of the phenomenon. The second case is advancing a much stronger and potentially more interesting claim: consciousness itself, as it naturally exists in the world, has a dimensional structure. Most models discussed here do not explicitly commit to one or the other use of the concept of dimensionality (although, for a notable exception, see section 3.2).

## 3 Dimensional models of consciousness

### 3.1 The bidimensional model of consciousness

As has been noted in the introduction, many theoretical texts on consciousness, both old and new, imply its dimensionality in some way. Possibly the most often implied dimension is the overall *quantity* or *level* of consciousness. There seems to be a strong pretheoretical intuition that one can be more conscious or less conscious (for an extended discussion of the semantics of “more conscious than” and “less conscious than” statements, see Lee, 2023).

Apart from being intuitively understandable and meaningful, the concept of a level of consciousness finds use in clinical practice, especially in neurology and psychiatry. Patients can be placed along a scale where normal, alert waking consciousness is at the top, deep coma at the bottom, and various intermediate conditions, such as the minimally conscious state, in between (Giacino et al., 2002).

In clinical contexts, consciousness is also described in the form of qualitative differences from normal waking consciousness. The paradigmatic example of such a state is delirium. At first glance, these departures from the norm of awake and lucid consciousness^2^ do not seem to form a clear dimension. However, some authors, such as those reviewed in this subsection, consider there to be a singular content- or awareness-related dimension to consciousness.

A bidimensional model with a *level* and a *content* dimension was described by, for example, Laureys (2005) or Monaco et al. (2005). In both texts, the fact that awareness is considered to be a gradable dimension is made clear by graphs representing it as an axis orthogonal to the level of consciousness. Vividness or emotional salience are pointed to as aspects of experience important for the qualitative dimension (Monaco et al., 2005). The level and content dimensions are also supposedly closely correlated in physiologically normal states like dreaming but dissociated in pathological conditions (Cavanna et al., 2011).

However, the *content* dimension of consciousness is also associated with the function of specialized modules providing the neural correlate for a specific kind of experience^3^ (Laureys, 2005; Monaco et al., 2005). Once again, the activity of these modules does not seem to form a single meaningful dimension comparable to the level of consciousness.

For further clarification, the Ictal Consciousness Inventory (ICI; Cavanna et al., 2008), developed by authors of the bidimensional model (Monaco et al., 2005), can be referred to. The ICI assesses consciousness during epileptic seizures on the dimensions of level and content. While there are other questionnaires describing altered states of consciousness using a number of dimensions (Pekala et al., 1986; Studerus et al., 2010; Ali et al., 2012), the ICI is directly related by its authors to the two dimensions of consciousness in their model. The questionnaire can be therefore interpreted as an attempt to psychometrically capture dimensions of consciousness itself, rather than merely describing the phenomenology of altered states of consciousness using dimensions.

The content subscale is said (Cavanna et al., 2008) to quantify the “vividness” of ictal experiential phenomena. The questions of the subscale ask about dream-like feelings, symptoms of derealization, sensed presence of another person, illusions, hallucinations, déjà vu, and the valence of emotions. The subscale was explained by a single factor with little cross-loading from the level subscale and had satisfactory item-to-total correlations and internal consistency, thus indicating that based on usual psychometric assumptions, the subscale most likely measures a single construct.

Still, the consistency of a psychometric measurement is by itself not an argument for the objective existence or theoretical meaningfulness of a certain construct. As the content dimension cannot be identified solely with vividness and its psychometric assessment includes heterogeneous, although correlated, psychopathology, it most likely cannot be conceptualized in any other way than as a multidimensional property in itself. This raises further questions about its subdimensions and the ways of aggregating them into a singular higher-order dimension of “overall quality” of consciousness that go currently unanswered by the literature.

While the bidimensional model of consciousness is widespread, mainly due to its clinical usefulness, its basic concepts remain in need of further explication. However, while the dimension of awareness appears more problematic, it has faced little scrutiny, with scientific attention concentrated mostly on the concept of an overall level of consciousness (Bayne et al., 2016a).

### 3.2 Quantity of consciousness in the Integrated Information Theory

Only one of the major theories of consciousness, the Integrated Information Theory (IIT), prominently features the concept of *quantity of consciousness*, equating it to the amount of integrated information (*Φ*) of a causal structure. This variable cannot be seen as identical to the intuitive or clinical conception of a level of consciousness, as will be seen in the overview of the theory below.

The IIT is closely related to its mathematical formalization, which cannot be presented thoroughly in the scope of this review. Any interested readers are therefore referred to the pivotal texts establishing the theory (Tononi, 2004; Oizumi et al., 2014; Albantakis et al., 2023). To ensure that all major claims of the theory are correctly represented, the quick summary below is guided by texts in which authors of IIT provide an abridged overview of the theory, omitting all but the most important concepts (Haun and Tononi, 2019; Tononi et al., 2022; Melloni et al., 2023).

The theory starts from phenomenology, arguing that certain claims about experience are immediately and irrefutably true for any possible form of consciousness. These are codified as the five axioms of IIT: intrinsicality, composition, information, integration, and exclusion. Experience is intrinsic when it is experienced from a subjective perspective, rather than only extrinsically, from the outside. Composition means that experiences have a structure, that they consist of phenomenal distinctions and relations, such as those between places in subjective space (Haun and Tononi, 2019). Information means that experiences are specific, rather than generic. Experience being integrated means that it forms a unified whole, being irreducible to its parts. Finally, the axiom of exclusion demands that consciousness is definite, having finite borders and a level of granularity.

From these axioms, five postulates that any physical systems must satisfy to be termed conscious are derived. Intrinsicality is taken to mean that the system must have cause-effect power upon itself, which necessitates causal feedback within the system, as purely feedforward structures only map inputs to outputs. Composition is translated into the system being a causal structure with subsets of units that are connected by (overlapping) causal relationships. The axiom of information means that the identified causal structure is specific, being composed of subsets of units in specific states. Integration means that the causal structure of the overall system cannot be reduced to the causes and effects of its parts. Lastly, exclusion means that the causal structure is specified by a definite set of units at a specific level of spatiotemporal grain. This ensures that there are no massive amounts of overlapping consciousnesses at once in any complex system, such as the human brain (Tononi et al., 2022).

The IIT then identifies elements of the causal structure with elements of experience: the specified causal distinctions and relations correspond directly to phenomenal distinctions and relations in conscious experience. A scalar measure of integrated information, of intrinsic cause-effect power over the substrate is introduced: *Φ*. In accordance with the axiom of exclusion, only the specific causal structure with a locally maximal value of *Φ* is conscious over any of its subsets (individual neurons) or supersets (groups of people).

Even though *Φ* has been given a mathematical definition (Tononi and Sporns, 2003; Albantakis et al., 2023), it can be realistically computed only for very simple models. Since its use is impractical for real neuroimaging data, it has been proposed that *complexity measures* provide a sufficient approximation of the brain’s *Φ* (Arsiwalla and Verschure, 2018; Sarasso et al., 2021). These measures estimate the complexity (for example, using Lempel-Ziv complexity) of a signal generated by the brain either at rest or when perturbed. Most relevant for IIT is the Perturbational Complexity Index (Casali et al., 2013), which was directly inspired by the theory. The index is calculated as the Lempel-Ziv complexity of EEG activity after perturbation by transcranial magnetic stimulation.

IIT’s concept of *Φ* has been criticized from various perspectives (Cerullo, 2015; Barrett and Mediano, 2019; Pautz, 2019), as has been the theory itself (e.g., Bayne, 2018; Doerig et al., 2019; Michel and Lau, 2020; Herzog et al., 2022; Fleming et al., 2023). However, for the purposes of our discussion about dimensions of consciousness, the most pivotal problem rests in the fact that *Φ* is not associated with any specific functional or phenomenal features of consciousness. In fact, one of the authors of the theory, Tononi (2014), readily acknowledges that even computationally very simple systems, such as grids of digital logic gates, can have arbitrarily large *Φ*, even when processing only a single binary input or when processing nothing at all. This is asserted as a counterintuitive prediction of the theory.

Additionally, IIT, as it is presented by its original authors (Albantakis et al., 2023), seems incompatible with any dimensionality of consciousness other than an overall *Φ* value, as this is a general characteristic of systems satisfying the postulates that the theory demands of the physical substrate of consciousness. Dimensional descriptions or structures derived from properties of human neurocognitive architectures, such as those referencing perception or metacognition, would be useful only for a subset (human brains) of all relevant systems (any physical system with feedback connections) and thus ultimately not central to IIT’s understanding of consciousness.

However, there may be potential in adopting a weaker version of the theory (Michel and Lau, 2020; Mediano et al., 2022; Leung and Tsuchiya, 2023), relaxing some of its metaphysical assumptions and treating integrated information as a useful correlate of consciousness, rather than as a necessary and sufficient condition for the presence of any kind of consciousness. Weak IIT is more likely to endorse some kind of multidimensionality of consciousness, as its scope and goals are more pragmatic and focused on biological neural systems, rather than supposedly universal in nature.

While the recent interest in complexity measures seems to be connected mostly with IIT, these results can be interpreted outside of the theory’s context, as will be demonstrated in the sections below (3.3, 3.5, and 4). Their success in differentiating states of consciousness also cannot be seen as a corroboration of IIT, as the theory is much broader and more ambitious in its scope. In fact, Koculak and Wierzchoń (2022b) advocate conceptually decoupling complexity measures research from IIT and adopting a neutral, less theory-driven stance on the relationship between consciousness and complexity.

### 3.3 The multidimensional account of global states

In their seminal paper, Bayne et al. (2016a) argue against the widespread conception that the property “being conscious” is gradable in the sense that some systems can be “more conscious” than others. They do so by proposing that the nature of consciousness, or rather of global states of consciousness, is multidimensional. The authors also outline two possible dimensions or families of dimensions: content-related and functional. The first is proposed to be associated with the gating of contents, which results in a different range of possible conscious experiences (e.g., a sedated person experiencing only fragmented perceptions). The second corresponds to the availability of conscious contents for different cognitive and behavioral systems, allowing them to drive intentional action and the reporting of experience.

Bayne et al. (2016a) define a *global state* of consciousness as the overall conscious condition of an organism. McKilliam (2020) provides an analysis of several possible more precise definitions of the term: a global state could be a kind of subjective experience itself, a certain structuring of experience, or the totality of everything experienced at some time point. In the end, McKilliam (2020) develops a conception of global states of consciousness as sets of functional capacities for different kinds of experience.

Following from the proposal of Bayne et al. (2016a), Bayne and Carter (2018) apply the argument against gradedness of consciousness to the psychedelic state, arguing that it deviates from normal waking consciousness in different directions on different putative dimensions. For example, while perception and imagination seem to become more rich and vivid, volition and attention tend to be impaired. The authors also note that the multidimensional approach seems at odds with both major theories of consciousness: the Global Workspace Theory (GWT; Baars, 2005) and IIT (Tononi et al., 2016). Both theories endorse a simple, unidimensional view on consciousness.

While the notion that global states of consciousness should be characterized dimensionally has been received well, specifics of the argument put forward in the above reviewed papers (Bayne et al., 2016a; Bayne and Carter, 2018) have been criticized (Fazekas and Overgaard, 2016; Fortier-Davy and Millière, 2020; Lee, 2023). Lee (2023) identifies two main lines of reasoning presented by Bayne et al. (2016a) against the gradability of consciousness: the Determinacy Objection and the Ordering Objection. As only the latter is strictly related to the multidimensionality of consciousness, the former will not be discussed here.

According to the Ordering Objection, if consciousness is multidimensional, then conscious states cannot be ordered, since a pair of states could be positioned oppositely on some of their constituent dimensions, thus making neither state “more conscious.” However, as has been pointed out (Fortier-Davy and Millière, 2020; Lee, 2023), this by itself does not make the relation “more conscious than” completely meaningless, as at least *some* pairs of states could possibly be unambiguously ordered. In fact, the mere multidimensionality of the property “being conscious” seems to tell us little about its orderability. Depending on the number of admissible dimension aggregation functions (D’Ambrosio and Hedden, 2023), a multidimensional property can be strictly unorderable (no aggregation of dimensions), vague (only some pairs of objects are ordered according to all possible aggregation functions), or even strictly orderable (only one possible aggregation function).

While Bayne et al. (2016a) and Bayne and Carter (2018) reject the possibility of a single gradable dimension of consciousness, elsewhere, Bayne et al. (2020a,b) argue for the use of unidimensional complexity measures as indications of the presence of islands of awareness. These metrics would possibly collapse several dimensions of consciousness into a single variable. In a recent review, Walter (2021) utilized similar reasoning when compiling evidence on correlates of complexity measures, arguing that complexity measures are possibly differentially sensitive to particular dimensions of the global state.

A problem raised by this approach is the fact that there is no clear criterion for what should count as a dimension of consciousness being reflected in the unidimensional marker and what should be considered a confound contaminating the metric. While, say, sensory richness could be plausibly seen as a dimension of consciousness (assuming the validity of the multidimensionality thesis), there is no way to decide whether lowered complexity in ADHD (Sokunbi et al., 2013) or anorexia nervosa (Tóth et al., 2004; Collantoni et al., 2020) represents a real attenuation of some dimensions of experience in these conditions or whether this is a limitation of the complexity measure itself as a proxy of consciousness. For example, brain signal complexity could be sensitive to structural alterations that have no bearing on the state of consciousness at all.

When discussing behavioral measures of consciousness, Klein and Hohwy (2015) outline a threefold distinction of what the relationship between a measure of consciousness and consciousness itself could look like. Either the measure is pure, directly capturing some measurable aspect of the phenomenon, such as a single dimension, or it is impure, reflecting several dimensions at once. The latter becomes much harder to avoid if the dimensions of consciousness are correlated. Or, most concerningly, it could be that the phenomenon itself is not measurable, a mongrel concept of some kind, perhaps incorporating both measurable and unmeasurable parts or even being logically incoherent.

A major problem of the approach advanced by Bayne et al. (2016a) is that there is currently no way to establish what is and what is not a dimension of consciousness. A multidimensional model of consciousness would need to rest on more precise empirical results and probably also make stronger theoretical commitments to resolve this issue. This would likely also necessitate clearing up the working concept of consciousness, instead of relying on definitions by example (Schwitzgebel, 2016) or broadly truistic and non-informative definitions, such as the famous “what-is-it-like” (Nagel, 1974).

### 3.4 The dimensions of conscious awareness

Responding to the article of Bayne et al. (2016a), Fazekas and Overgaard (2016) agree with their proposal of multidimensional global states, but they argue that this account omits another way in which consciousness is dimensional: the conscious awareness of contents coming in degrees.^4^ According to Fazekas and Overgaard (2016), this gradable property of consciousness resides in how much and in what way is the quality of the perceived stimulus degraded in contrast to normal, clear perception.

Three dimensions of the quality of conscious awareness have been proposed: intensity, specificity, and temporal stability (Fazekas and Overgaard, 2018; Fazekas et al., 2020). These dimensions are then said to have neural counterparts: the amplitude, precision (attenuated variance), and duration of the neural code, respectively. In further work, these dimensions have been applied to both online and offline perceptual experience. For example, it has been claimed that dream content can be described along the dimensions of intensity, specificity, and stability (Nemeth and Fazekas, 2018; Fazekas et al., 2019; Fazekas, 2024).

Bayne et al. (2016b) disagree with Fazekas and Overgaard (2016) on the basis of the argument that while consciousness can differ in its content, it does not differ in the fact that the subject is conscious; one cannot have “more of” a subjective point of view. This is the Determinacy Objection, which has been analyzed in detail and rebutted by Lee (2023). In the view of Bayne et al. (2016b), therefore, a subject experiencing a degraded perception should be understood as the result of variation in the quality of perceptual content, not as variation in consciousness or any of its properties.

Anzulewicz and Wierzchoń (2018) instead dispute the simplicity of the relationship between neural code and subjective features. They stress that attention modulates perception in disparate ways at different stages of processing, as well as the influence of factors other than sensory information or attention. These include, for example, the effects of context, prior experience, or expectations.

As it stands, the model of Fazekas and Overgaard (2016, 2018) is appealing in its simplicity and straightforward relationship between features of experience and neural activity. However, it is also limited in scope and says little about the neural or cognitive processes involved in generating conscious perceptual experience. Another possible point of contention is the relation of “degraded” perceptions to “normal” perceptual experience, although the proposed dimensions could be easily extended to accommodate a range of “higher” forms of experience. Certain altered states of consciousness, for example, could be said to produce experiences more intense than normal perception.

### 3.5 Four-dimensional graded consciousness

Another view on the dimensions of consciousness is the so-called “four-dimensional graded consciousness” introduced by Jonkisz et al. (2017). In earlier works, Jonkisz (2012) argued that there are four major criteria commonly used to delimit the concept of consciousness. The epistemic criterion distinguishes the subjective or phenomenal from objective or access consciousness. The semantic criterion represents the level of abstraction from immediate sensorimotor awareness, in higher order mental representations, for example. The physiological criterion differentiates wakefulness, sleep, and altered or impaired states of consciousness. And finally, the pragmatic criterion concerns the sources and usefulness of consciousness, as well as the specifics of the exact system that is conscious, distinguishing, for example, the normal awake conscious state and cortical islands of awareness causally isolated from the outside world.

Over time (Jonkisz, 2015, 2021; Jonkisz et al., 2017), these criteria were developed into fundamental features of a singular concept of consciousness in the form of gradable dimensions: phenomenological, semantic, physiological, and functional. While authors of the theory (Jonkisz, 2015) agree with IIT on consciousness being an informational property, they stress the importance of consciousness existing, as far as is currently known, exclusively in evolved biological systems. They thus avoid the attribution of consciousness to photodiodes or XOR gates based on pretheoretical intuitions, while IIT treats the assumed ubiquity of consciousness in non-living systems simply as a counterintuitive prediction.

The authors also put forward possible operationalizations for each of the four putative dimensions of consciousness. The first two dimensions, phenomenological and semantic, are reflected in subjective measures of consciousness. Jonkisz et al. (2017) hypothesize that directly asking subjects to report on their experience (for example, by using the Perceptual Awareness Scale; Ramsøy and Overgaard, 2004; Overgaard and Sandberg, 2021) is more closely related to varying phenomenal quality, while using confidence ratings as an indicator of consciousness may tap into more metacognitive processes, indicating higher levels of abstract, semantic experience.

Complexity measures, in their view, map the physiological dimension of consciousness instead of overall quantity. Lastly, the functional dimension of consciousness is not assigned to any experimental paradigm in psychology or neuroscience but is instead related to the “Bayesian brain” hypothesis (Friston, 2012; Seth and Friston, 2016). The functionality of consciousness may therefore be a dimension that increases with the brain’s capabilities to arrive at efficacious predictions about the world.

The four-dimensional model of Jonkisz et al. (2017) is more concrete than the multidimensional account of Bayne et al. (2016a), while also avoiding the strong axiomatic commitments of IIT (Tononi et al., 2016). The connections made between dimensions of consciousness and specific behavioral or neural measures can be seen as a major strength of the proposal. On the other hand, a weak point remains in the step from the four outlined dimensions being considered criteria for defining consciousness (Jonkisz, 2012) to becoming dimensions of consciousness itself (Jonkisz et al., 2017). The authors do not provide a strong argument for their list of dimensions being exhaustive, thus not resolving the problem of specifying a theoretically sound method for determining the number and nature of variables that can be understood as dimensions of consciousness.

### 3.6 The Temporo-Spatial Theory of Consciousness

The last theory of dimensionality of consciousness that will be presented in this review was developed from the bidimensional model of level and content presented in section 3.1. Northoff (2013) postulated, based on the influence the brain’s baseline activity fluctuations have on contents entering consciousness, a third dimension of consciousness: form. This dimension consists of embedding the stimulus-related neural signals into the context of intrinsic resting-state activity by mechanisms such as phase locking (Sauseng and Klimesch, 2008).

This tridimensional model was soon replaced by the four dimensions of the Temporo-spatial Theory of Consciousness (TTC) proper: the overall level or state, the content or form, phenomenal features, and global cognitive access (Northoff and Huang, 2017; Northoff and Zilio, 2022b). Each of the dimensions was assigned a specific neural mechanism depending solely on the temporospatial dynamics of brain activity. The overall level of consciousness was identified with scale-free dynamics and a small-world topology of functional networks (“nestedness”). The dimension of content or form, which resulted from merging the two corresponding dimensions in the tridimensional model, is equated to “alignment,” meaning the integration of stimulus-related activity into intrinsic baseline fluctuations.

The mechanism underlying the phenomenal dimension of consciousness was termed “expansion” and related to the higher amplitude and longer duration of stimulus-generated signals that end up being consciously processed. Lastly, global cognitive access has been associated with a mechanism of “globalization,” which is the continuation of expansion by recruitment of higher brain regions, enabling the reporting of conscious experience.

Each of the dimensions can be seen as a prerequisite for the next: appropriate level of consciousness is necessary for alignment, which is important for perceptual contents entering consciousness. This is evidenced by the phenomenon of “phase preference” (Northoff and Huang, 2017), where the pre-stimulus phase of brain oscillations affects whether a near-threshold percept reaches consciousness (Mathewson et al., 2009; Glim et al., 2020). Aligned stimulus-related activity then needs to be spatially and temporally expanded before reaching more frontal brain areas necessary for its reportability (Boly et al., 2017; Odegaard et al., 2017).

The TTC claims to explain the unity of the stream of consciousness and its relation to time better than other theories of consciousness (Northoff and Zilio, 2022b). While it has some similarities with IIT, such as the focus on low-level and general features of neural activity (or causal structures), it distinguishes itself by moving to an even more fundamental level of explanation: from integrated information to the brain’s time and space (Northoff and Huang, 2017). Elsewhere, authors of the theory argue that IIT can be subsumed into the TTC framework (Northoff and Zilio, 2022a), as can other theories of consciousness (Northoff and Lamme, 2020).

A strength of TTC is its utilization of empirical findings rarely considered by other theories, such as the influences of pre-stimulus activity on conscious awareness. However, being a broad and ambitious theory, the exact nature of its claims remains to be thoroughly explicated in future work. This includes the definition and possibilities of measurement of the proposed dimensions, as well as the question of to what degree are they simply useful descriptions or fundamental features of the phenomenon of consciousness. Finally, as TTC is currently a relatively marginal theory of consciousness, especially compared to GWT and IIT, it has not received much thorough criticism from other researchers of consciousness.

## 4 Empirical research on the dimensions of consciousness

While all of the reviewed models draw on a body of empirical work within the field of consciousness research, there are currently very few research papers that are specifically aimed at studying, in some way, the dimensions of consciousness. The few research directions that have been proposed or pursued so far are briefly reviewed in this section.

One possible approach involves using subjects’ own introspective reports in the form of questionnaire responses. The ICI (Cavanna et al., 2008), as a method intended to place patients’ experiences during epileptic seizures on the two dimensions of the bidimensional model, has already been reviewed in section 3.1. However, there is also a group of questionnaires assessing altered states of consciousness that have seen extensive use in research (Schmidt and Berkemeyer, 2018), some of which describe altered states using dimensions.

One of these questionnaires is the Phenomenology of Consciousness Inventory (Pekala et al., 1986) whose authors have consistently claimed that the questionnaire’s dimensions correspond to dimensions of conscious experience or consciousness itself (Pekala and Levine, 1982; Pekala and Kumar, 2007). However, while some dimensions of the questionnaire could very plausibly be considered dimensions of experience in general, such as affect or arousal, others relate the altered state to normal awake consciousness by directly asking participants to compare their experience to what is ordinary or usual for them.

A problem for all self-report measures, if they are meant to provide evidence for the dimensionality of consciousness, is the dependence on retrospective introspection. Subjects in most unusual states of consciousness are unable to give concurrent reports on their own experience. The methods therefore presuppose that all relevant features of the experience are encoded into memory and then recollected successfully. More fleeting forms of experience are unlikely to be described using this method, as are certain pathological states which would be of interest to researchers of consciousness, but from which subjects may emerge only after a long time or never at all.

Another possibility for finding the dimensions of consciousness comes from the use of complexity measures. The before-mentioned approach of Walter (2021) lies in analyzing the correlates of these measures and treating them as candidate dimensions of consciousness. This method directly follows from the proposal of Bayne et al. (2016a, 2020b), but, as we have seen, different groups of authors attribute different meanings to complexity measures.

The problem with this approach has also been mentioned before in this review: complexity measures should correlate with dimensions of consciousness, as well as with confounding variables. These could be biological or physical in nature, such as structural changes not affecting the function of the brain, but also psychological, including differences in cognitive functions that have no bearing on the state or content of consciousness.

A different branch of research that has been associated with the dimensions of consciousness by some of the authors reviewed above (Bayne et al., 2017; Walter, 2021) is the multidimensional assessment of disorders of consciousness. For example, Sergent et al. (2017) have developed an EEG protocol made up of eight tasks that require no behavioral response, such as global incongruence detection or own name perception. Combining the information from EEG activity in response to each of these tasks provided improved classification of patients either in the vegetative or the minimally conscious state, as well as an individualized map of residual cognitive capacities in each of the subjects, unlike the unidimensional complexity measures.

The efficacy of the multidimensional assessment of disorders of consciousness could be seen as corroboration of the thesis that consciousness is multidimensional, as the responses to each task likely draw on different subsets of cognitive functions of the subject. However, it is not clear what the relationship between these tasks and dimensions of consciousness itself should be, or even, in fact, if all of them correspond to variation in experience. This kind of research, I argue, therefore runs into similar difficulties as the correlational approach presented above.

Finally, one unique approach has been recently advanced by Huang et al. (2023). Using dimensionality reduction on resting state fMRI data, they identified three neurofunctional gradients that were differentially affected by the administration of propofol or ketamine and in pathological conditions like unresponsive wakefulness syndrome and schizophrenia. The authors directly relate these gradients to dimensions of consciousness, interpreting them as representing awareness (impairment of which results in dissociative states), sensory organization, and arousal.

While the use of neuroimaging data and data-driven dimensionality reduction techniques avoids many of the pitfalls of the previously presented research, this route is still not without its problems. Use of fMRI data presupposes that the kind of neural activity that is relevant for dimensions of consciousness can be localized on this temporospatial resolution. It could be that fast oscillations or activation on the meso- to microscopic level are better related to some dimensional description of consciousness. Furthermore, normal waking consciousness was represented only by the “resting state with eyes closed” condition, which corresponds only to a restricted and unusual kind of waking experience (Koculak and Wierzchoń, 2022a).

## 5 Discussion

A variety of views on the ways in which consciousness can be thought of as dimensional have been critically reviewed. A common issue of the multidimensional models seems to be the way in which the correct dimensions of consciousness can be disambiguated from the range of possibilities, including biological confounds and psychological constructs unrelated to consciousness. The importance of this question, in my opinion, precedes even the problem of whether dimensions of consciousness can be aggregated to compare different states on their overall level of consciousness.

Resolving this issue would be a substantial contribution to a set of proposals in the science of consciousness that interrogate whether certain neurological or psychiatric conditions correspond to alteration in the usual state of consciousness. The possibility of such altered forms of consciousness has been considered, among possibly others, in the case of depression (Whiteley, 2021), autism (Yatziv and Jacobson, 2015), and schizophrenia (Bob and Mashour, 2011; Bob, 2012). A robust multidimensional model of consciousness would also allow inferences about alterations of experience in other conditions, such as interictal epilepsy, personality disorders, or after a head injury. On the other hand, a well-formulated model should also avoid overinclusivity in what it considers a dimension of consciousness and which experiential differences cannot be treated as changes in consciousness itself.

More controversially, changes in the dimensions of consciousness could also be associated with intelligence.^5^ Its assessment routinely includes cognitive functions related to consciousness, such as the span of working memory, the content of which is often equated with the total content of consciousness, or detection of details, possibly reflecting a richer phenomenal scene or a higher level of semantic abstraction of the perceived objects. Evidence on the exact nature of the relationship between intelligence and complexity measures is currently mixed (Saxe et al., 2018; Dreszer et al., 2020; Wang, 2021; Thiele et al., 2023).

However, if a clear positive correlation between intelligence and a putative dimension or dimensions of consciousness were to be established in the future, the interpretation and presentation of these results would need to be treated extremely carefully. Given the historical issues with intelligence testing, validating the view of less intelligent people as “less conscious” and the possibility of “ranking” people according to the degree to which they are conscious could have even more dire consequences than misuse of intelligence testing by itself, especially since moral status is often closely tied to consciousness (Jeziorski et al., 2023; Mazor et al., 2023).

If a multidimensional model of consciousness is to be widely accepted, though, it is very likely that it would have a hierarchical structure, similar to intelligence in the Cattell–Horn–Carroll model (Schneider and McGrew, 2012), with each higher-order dimension, such as quantity or sensory richness, itself being a multidimensional property. The idea that some of their putative dimensions could really be families of dimensions was already advanced by Bayne et al. (2016a), and one recent theoretical proposal framed consciousness in direct comparison to hierarchical models of intelligence (Kent et al., 2019). This dimensional structure would also be compatible with the understanding of the dimension of content or awareness in the classic bidimensional model as multidimensional.

A notable feature of the reviewed literature is the absence of major theories of consciousness other than IIT, chiefly GWT (Baars, 2005). The missing representation of this theoretical space may be partly explained by the association of complexity measures with IIT. However, GWT is not conceptually incompatible with the dimensional description or structure of consciousness, and neither is it, as has been recently argued (Farisco and Changeux, 2023), incompatible with the use of complexity measures to characterize states of consciousness.

Future work in this specific area of consciousness research should focus on approaching the main issues identified above: the problems of dimensions selection and dimension aggregation. A promising avenue for this sort of clarifying effort seems to lie in the more atheoretical works of Bayne et al. (2016a) or Jonkisz et al. (2017). Dimensional models tied to a larger theory of consciousness with non-trivial axioms and philosophical commitments seem to be less well-suited. However, proceeding with a weak version of the theory (such as weak IIT; Mediano et al., 2022) might be promising, as this allows retaining the language and conceptual tools of the theory while relaxing the more restrictive assumptions.

Furthermore, a common problem of theories of consciousness, which pervades this topic as well, is the significant intangibility of most proposals and the resulting distance of theoretical concepts from existing psychological and neuroscientific methods or data. This distance allows very disparate predictions to be drawn from the same hypotheses, undermining the requirement of falsifiability that is necessary for any theory to be considered scientific (Fleming et al., 2023; Lau, 2023). Claims concerning dimensions of consciousness therefore need to be more specific and testable using currently available methods to make a difference in the broader discussion about the nature of consciousness.

Finally, it may be speculated that if multiple well-founded accounts of the dimensionality of consciousness emerge in the future from the current state of the field, further work could be conducted identifying the points of friction between their explanations and resolving these with adversarial collaborations.

## Author contributions

JP: Conceptualization, Writing – original draft.

## Footnotes

^1^It should be noted that the present analysis is closely related to the concept of *levels* or *degrees* of consciousness, which can be seen as a special case of the dimensionality of consciousness, namely unidimensional consciousness with a dimension of overall quantity.

^2^Symptoms of qualitative disorders of consciousness can include illusions, hallucinations, confusion, altered perception of time, dissociation, and dream-like qualities.

^3^For example, the fusiform face area can be thought of as instantiating a module for the experience of seeing a face.

^4^It is worth mentioning that in their original response to Bayne et al. (2016a), the construct that is described as dimensional is simply “consciousness” (Fazekas and Overgaard, 2016), while in later works this target phenomenon is termed “awareness” (Fazekas and Overgaard, 2018) or “conscious experiences” (Fazekas et al., 2020; Fazekas, 2024). For the purposes of this review, only the term “conscious awareness” is used to distinguish the explanandum of this model from the global state of consciousness (cf. Bayne et al., 2016a).

^5^As has been pointed out by an anonymous reviewer, the relationship between consciousness and intelligence is much more complicated than what can be reflected within the scope of this review. In the broadest sense of the term, consciousness can encompass highly complex and flexible mental processes, such as creativity or mindful states. However, since these functions of consciousness are rarely considered by the models reviewed here (possibly with the exception of the functional dimension in the model of Jonkisz et al., 2017), these implications of the dimensional conception of consciousness are left here unexplored. Future work should, however, address this broader question of the functional role of consciousness above that of intelligence.
